# Effects on voice hearing distress and social functioning of unguided application of a smartphone app — A randomized controlled trial

**DOI:** 10.1016/j.invent.2024.100717

**Published:** 2024-01-26

**Authors:** Alyssa Jongeneel, Philippe Delespaul, Nynke Tromp, Dorien Scheffers, Berber van der Vleugel, Paul de Bont, Martijn Kikkert, Carlos F. Croes, Anton B.P. Staring, Heleen Riper, Mark van der Gaag, David van den Berg

**Affiliations:** a Department of Clinical, Neuro- and Developmental Psychology, Amsterdam Public Health Research Institute, Vrije Universiteit Amsterdam, Netherlands; b Research Centre, Parnassia Psychiatric Institute, Zoutkeetsingel 40, 2512 HN Den Haag, Netherlands; c School of MentalHealth and NeuroSciences, Maastricht University, Maastricht, Netherlands; d Mondriaan Mental Health Centre, Maastricht, Heerlen, Netherlands; e Department of Industrial Design, Delft University of Technology, Landbergstraat 15, 2628 CE Delft, Netherlands; f Community Mental Health Service, GGZ Noord-Holland Noord, Alkmaar, Netherlands; g Viersprong Institute for Personality Disorders, Amsterdam, Netherlands; h Department of Early Intervention Psychosis, Mental Health Organisation GGZ Oost Brabant, Land van Cuijck en Noord Limburg, Boxmeer, Netherlands; i Arkin Research, Arkin, Amsterdam, Netherlands; j GGz Centraal Mental Health Institution, Amersfoort, Netherlands; k Altrecht Psychiatric Institution, Utrecht, Netherlands; l Department of Research and Innovation, GGZ InGeest Specialized Mental Health Care, Amsterdam, Netherlands

**Keywords:** Voice hearing, Voices, Auditory verbal hallucinations, Mhealth, ESM, Psychosis

## Abstract

**Background:**

Temstem is a smartphone app developed with and for clinical voice hearing individuals with the aim to reduce their voice hearing distress and improve social functioning.

**Methods:**

A randomized controlled trial with adult outpatients suffering from distressing and frequent auditory verbal hallucinations (AVH) was conducted. Participants were randomized to unguided ‘Temstem+AVH monitoring’ or unguided ‘AVH monitoring only’ (control condition). Assessments were performed at baseline, post-intervention (week 5–6), and follow-up (week 9–10). Primary outcomes were voice hearing distress and social functioning, as measured with Experience Sampling Method (ESM), consisting of multiple daily questionnaires during six days. In addition, voices and mood were self-monitored with help of a daily reflective questionnaire. Analyses were linear regression models (intention-to-treat).

**Results:**

44 Participants were allocated to Temstem and 45 to the control condition. No significant differences between the groups were found on both primary outcomes.

**Conclusion:**

Our results do not support the effectiveness of stand-alone use of Temstem versus symptom monitoring on voice hearing distress or social functioning in voice hearing individuals. In order to potentially improve effectiveness of an mHealth tool in a population of people with frequent and distressing voices, we recommend to involve persons with lived experience in all stages of development and research; to thoroughly test the (technological) usability before performing an RCT; to test whether guidance of a therapist is needed to optimize effectiveness; and to provide prompts to remind the user to actually use the tool.

## Highlights

• The Temstem app is a coping tool for persons with distressing voices.
• Stand-alone Temstem did not reduce daily life distress.
• Stand-alone Temstem did not improve social functioning.
• Persons with lived experience should be continuously involved.
• Guided Temstem might encourage voice hearers to use the app.

## 1 Introduction

Voice hearing, or auditory verbal hallucinations (AVH), is most prevalent in persons suffering from a psychotic disorder, with a prevalence rate of about 75 % in persons with lifetime schizophrenia (Bauer et al., 2011). However, it also occurs in over 65 % of the persons with bipolar disorder (Baethge et al., 2005), 50 % of those with post-traumatic stress disorder (Anketell et al., 2010), 40 % of those with depressive disorder (Baethge et al., 2005), and 27 % of those with borderline personality disorder (Niemantsverdriet et al., 2017). Thus, voice hearing is a highly prevalent transdiagnostic phenomenon.

Hearing voices can be a very distressing experience with major impact on peoples wellbeing and functioning. Voice hearing is associated with increased rates of anxiety, depression (de Leede-Smith and Barkus, 2013), disruption of life (Slotema et al., 2012), and suicide attempts (Fujita et al., 2015). The current guideline treatments in many countries exist of antipsychotic (AP) medication and cognitive behaviour therapy for psychosis (CBTp). A systematic review and meta-analysis reported that antipsychotic medication significantly decreased positive symptoms in persons with psychosis compared to a placebo (g = 0.45) (Leucht et al., 2017). In addition, Turner, Burger, Smit, Valmaggia, & van der Gaag (Turner et al., 2020) showed in their meta-analysis that CBTp was, compared to a control condition, significantly more effective in reducing auditory hallucinations in psychosis (g = 0.34, *P* < .01) and according to the systematic review and meta-analysis of Bighelli et al. (Bighelli et al., 2018), about 50 % of patients reached at least a small (20 %) reduction of positive symptoms by CBTp and about 25 % of patients reached 50 % symptom reduction. This is comparable to antipsychotic medication (Leucht et al., 2017). Nonetheless, there is room for improvement and broadening the range of interventions.

Digital interventions are emerging and might offer new opportunities in the treatment of psychosis. Smartphone applications (apps) have the advantage that they can be used real time at the moment that an individual experiences voices. While a part of app interventions involve varying degrees of guidance by a therapist (i.e. blended), other apps can be used unguided (i.e. stand-alone). Most interventions in psychosis rely on the clinician-patient interaction, which is expensive. An unguided app can be widely deployed, and then even small effects may lead to important health gains and even be cost-effective.

An example of an unguided app for people suffering from psychosis, is Actissist (Bucci et al., 2015). Users fill in questions in the app and based on the answers, they receive normalizing messages or CBTp based strategies. The app also includes information, activities, recovery stories and more. In a pilot study with 36 persons with psychosis, it was observed that Actissist (*n* = 24), compared to a group using a symptom monitoring app (*n* = 12), significantly decreased negative symptoms (d = −0.85) and total psychosis symptoms (d = −0.86), although these effects did not sustain at 22-week follow-up (Bucci et al., 2018). An example of an app intervention for voice hearing whereby participants receive guidance by a therapist, is SAVVy. In SAVVy (Smartphone-Assisted coping focused interVention for Voices; (Bell et al., 2018; Bell et al., 2020), voice hearers fill in questionnaires in the app and receive individualized coping strategies. Within this protocol, patients have four face-to-face sessions with a therapist. The pilot trial did not find a significant difference between SAVVy (*n* = 17) and TAU (n = 17) on severity of voice hearing (Bell et al., 2020).

To further add to the knowledge base on the effectiveness of apps for distressing voice hearing, this current study focusses on an (unguided) app intervention: Temstem (‘Tame the Voice’). Temstem is a coping tool, developed for and in co-creation with individuals suffering from voice hearing (Jongeneel et al., 2018) (see Appendix A for more details on the co-creation process). As mentioned before, voices can be very distressing and may disrupt someone's life. By coping with voices ‘in the moment’, i.e. at the moment the voices occur, voice hearing distress can be directly (temporarily) decreased. Consequently, a person might endure a certain difficult situation (e.g. visiting the supermarket while hearing voices) or the threshold to engage in certain (social) situations might become lower.

Temstem consists of two language games. By means of two functions, Temstem aims to reduce voice hearing distress and to improve social functioning; ‘Silencing’ is developed to improve control over voices by incompatible language behaviour, and ‘Challenging’ to reduce vividness and emotionality of voices by dual tasking. In Silencing, individuals actually use language expression while hearing voices, which is known to (temporarily) suppress voices in most people (Bick and Kinsbourne, 1987; Erickson and Gustafson, 1968; Green and Kinsbourne, 1989). In the Challenging function, individuals are asked to recall a negative voice hearing statement, while playing a language game (‘dual tasking’). Research showed that dual tasking reduces the emotionality, vividness, and credibility of voice memories (Jongeneel et al., 2020; Matthijssen et al., 2019).

Besides these two functions, the avatar ‘Tim’ provides frequent positive and constructive feedback to the user. In the Challenging function, Tim provides feedback based on this selected cognitive affective domain that is triggered by the voices (e.g., when a user indicates feeling powerless, Tim emphasizes that the person is strong). Voice hearing can trigger feelings of depression and self-esteem issues, and these ‘counter-themes’ might be useful in decreasing feelings of depression and improving self-esteem (Van Der Gaag et al., 2012).

In a naturalistic study whereby data of 1.048 Temstem users was analyzed, we observed that users experienced a significant decrease in voice hearing distress after a Silencing session (d = 0.49), and a significant reduction of emotionality (d = 0.74) and vividness (d = 0.71) of voice memories after a Challenging session (Jongeneel et al., 2022). There was no control condition in this study; therefore, it is not certain that the reductions in voice hearing distress and emotionality and vividness of voice memories were caused by Temstem. Still, these results appear promising and we expected Temstem to be effective in reducing voice hearing distress in a sample of persons with distressing and frequent voice hearing experiences. This paper presents the primary outcomes of the Temstem Randomized Controlled Trial.

Hypothesis 1a Momentary distress will decrease more in the Temstem condition than in the control condition, as measured over multiple daily life assessments by Experience Sampling Method (ESM).

Hypothesis 1b Subjectively experienced distress caused by AVH will decrease more in the Temstem condition than in the control condition, as measured by daily reflective monitoring.

Hypothesis 2a Social functioning will improve more in the Temstem condition than in the control condition, as measured over multiple daily life assessments by ESM.

Hypothesis 2b Subjectively experienced negative impact of AVH on social functioning will decrease more in the Temstem condition than in the control condition, as measured by daily reflective monitoring.

This paper also includes results of secondary outcomes (hypotheses 3–7) as defined in our protocol paper (Jongeneel et al., 2018). Details of these outcomes can be found in Appendix B.

## 2 Material and methods

### 2.1 Design and participants

The Temstem trial is a single-blind randomized controlled trial with two conditions: ‘Temstem and AVH monitoring’ versus ‘AVH monitoring only’ (hereafter called respectively ‘Temstem’ and ‘control’). The adult (18+) participants were in care for severe mental illness in one of the twelve participating Dutch specialized mental health organizations. Full details of the study protocol have been published (Jongeneel et al., 2018).

Inclusion criteria were, irrespective of diagnosis, the presence of distressing AVHs for longer than one month, and experiencing the AVHs during a minimum of 4 days a week in at least three out of the last four weeks. Exclusion criteria were insufficient mastery of the Dutch language, current involuntary admission in a closed ward, an (estimated) IQ of below 70, modifications in antipsychotics and/or antidepressants during the last month, currently receiving CBT for AVHs, unwilling or uncapable to learn to use a smartphone, and intensive previous or present use of Temstem.

The trial design was approved by the Medical Ethics Committee of the VU University Medical Centre (METC number: 2015.435/NL53684.029.15) and was registered online (ISRCTN75717636). The authors assert that all procedures contributing to this work comply with the ethical standards of the relevant national and institutional committees on human experimentation and with the Helsinki Declaration (2008).

### 2.2 Procedure

**Fig. 1:**
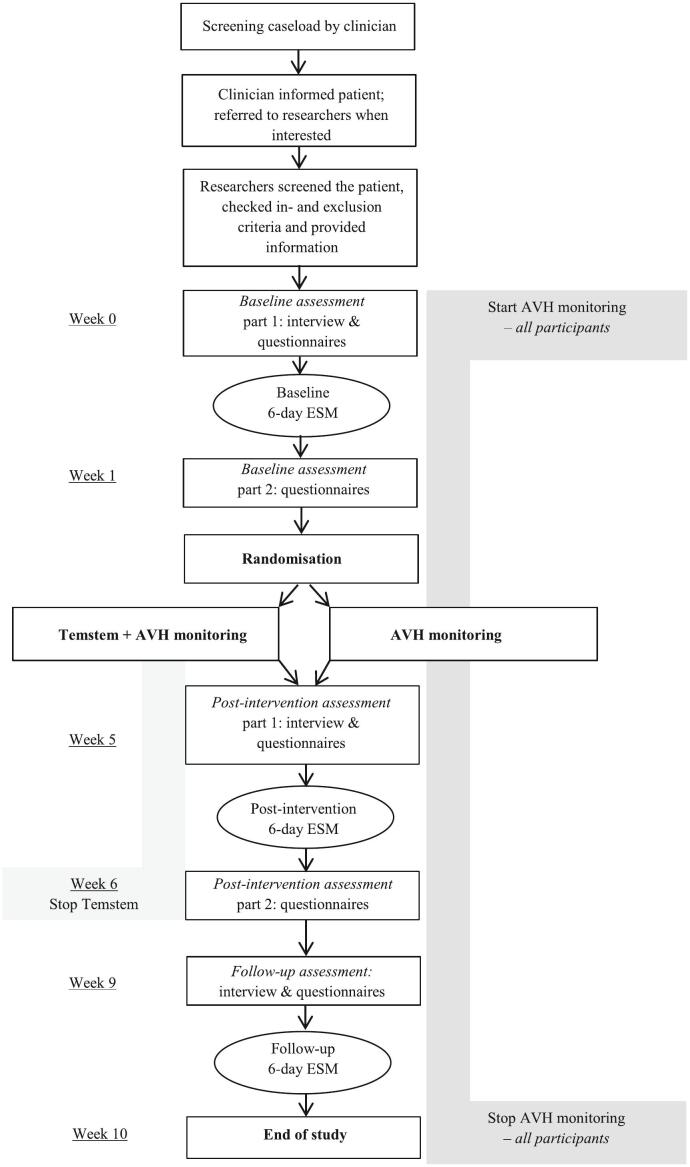
Flowchart assessments.

Therapists of the involved mental health care institutions provided a 1-min screener to patients in order to check their eligibility and willingness to participate (See Fig. 1). The therapists then send the screener to the researchers. The researchers checked the in- and exclusion criteria and provided more information. After at least one week for considering participation, the patient was invited for the first appointment during which informed consent was signed and the baseline measurements were started.

During the face-to-face baseline assessment, a clinical interview and several questionnaires were conducted (see 2.6.3 Interview and questionnaires). All participants received a smartphone with the installed Experience Sampling Method (ESM) app (PsyMate) which they used for 6 consecutive days. They also started monitoring their voices with a short daily questionnaire (daily monitoring) via the PsyMate app, which was pursued for the duration of the study period. After completing at least 25 out of 60 ESM questionnaires, the remainder of the baseline assessment was performed. After finishing the baseline assessment, participants were randomized to either the Temstem or the control condition. Participants allocated to the Temstem condition received face-to-face instructions on how to use Temstem and briefly practised with the app.

After four weeks, the post-intervention assessment was performed. It consisted of a clinical interview and questionnaires; a 6-day ESM period; and several questionnaires. Hereafter participants in the Temstem condition stopped using Temstem. This assessment procedure was repeated three weeks later for follow-up assessment. After the third ESM period the study was concluded.

### 2.3 Interventions

#### 2.3.1 Temstem

Participants in the Temstem condition used Temstem autonomously for five weeks. After one week they were contacted by phone by a research assistant to check utilization capability and possible obstacles. In addition, participants kept monitoring their voices daily by using the ESM app.

#### 2.3.2 Control

Participants in the control condition continued monitoring their voices daily with the ESM app.

### 2.4 Technical procedure

In case of failure or problems with the Temstem and/or ESM app, an unblinded research assistant called or visited the participant to solve the issue. In case of insurmountable technical failure of the phone, a new phone was given to the participant. Technical problems with the apps were registered and reported in Appendix D.

### 2.5 Randomisation and masking

Block randomisation was used to assign participants to the Temstem or control condition via an online randomisation program (http://www.randomizer.org). Participants were randomized directly after baseline assessment. The research assistant conducting the baseline assessment was informed about the allocation, in order to give instructions how to use Temstem (if applicable), and did not perform any follow-up assessments.

### 2.6 Measures

#### 2.6.1 Experience sampling method

The PsyMate app (www.psymate.eu) was used for ESM measurement. At baseline, post-intervention, and follow-up, participants got prompted ten times a day for six consecutive days with a 45-item questionnaire. This questionnaire involved questions about symptoms, mood, and context. For an overview of questions, see Jongeneel et al. (Jongeneel et al., 2018).

The outcome variable for hypothesis 1a, momentary distress, was obtained by calculating the mean of the following negative affect items that were scored on a 7-point Likert scale (1 = not at all to 7 = very): I feel insecure/sad/lonely/anxious/irritated/relaxed (reversed).

The outcome variable for hypothesis 2a, social functioning, was measured as follows:

1. The categorical activity question: ‘What am I doing, just before the beep?’. The categories resembling ‘passive’ activities were: resting, eating/drinking, relaxing-passive, and/or doing nothing; they were scored a 0. The categories resembling ‘active’ activities were: work/school/study/homework, household/groceries, traveling/transport; they were scored a 1. In addition, the categories hygiene/selfcare, relaxing-active, and/or doing something else, were scored a 1 when individuals additionally rated a 3–7 on a 7-point Likert scale of the question ‘I feel like I'm being active’; if score 1 or 2 was given, these categories were also scored as 0.
2. The categorical social company question: ‘Who am I with?’. The categories: being alone or with a pet, resembled score 0. The categories: being with partner, fellow residents, family, friends, colleagues, acquaintances, strangers and/or others, were scored as 1 when individuals additionally rated a 3–7 on the 7-point Likert scale of the question: ‘We are doing something together’; if score 1 or 2 was given, these categories were also scored as 0.
3. When both the activity and social company question scored a 0, the social functioning variable was a 0; when one or both were scored by a 1, social functioning was scored as a 1.

Secondary hypotheses were also measured by ESM, see Table 1 Appendix B.

**Table 1:** Baseline characteristics.

Characteristic	Temstem condition (***n*** = 44)	Control condition (***n*** = 45)
Female, no. (%)	28 (63.6)	21 (46.7)
Age in years: mean (SD)	44.68 (12.25)	41.29 (10.25)
Non-Dutch origin^a^, no (%)	13 (29.5)	19 (42.2)
Education, no (%)		
None or primary	4 (9.1)	4 (8.9)
Vocational	30 (68.2)	29 (64.4)
Secondary	4 (9.1)	6 (13.3)
Higher	6 (13.6)	6 (13.3)
Primary DSM-IV-TR chart diagnosis, no (%)		
Schizophrenia	14 (31.8)	25 (55.5)
Schizoaffective disorder	9 (20.5)	1 (2.2)
Psychotic disorder NOS	8 (18.2)	10 (22.2)
Post-traumatic stress disorder	5 (11.4)	3 (6.7)
Borderline personality disorder	4 (9.1)	1 (2.2)
Mood disorder	1 (2.3)	4 (9.1)
Other^b^	3 (6.8)	1 (2.2)
Medication use, no (%)		
Antipsychotics	38 (86.4)	41 (93.2)
Antidepressants	22 (50.0)	16 (35.5)
Voice hearing duration, in years: mean (SD)	15.8 (11.8)	16.4 (11.4)

Note: NOS = not otherwise specified.

a Non-Dutch origin is defined when the participant or one or both of his/her parents was/were born in another country than the Netherlands.

b Other consists of: anxiety disorders, dissociative identity disorder, and other personality disorders.

#### 2.6.2 Daily reflective monitoring

Every evening during the whole ten-week study period, participants completed a short questionnaire on the PsyMate app to reflect on their voices, feelings, functioning, and more.

The outcome variable for hypothesis 1b, subjectively experienced distress caused by AVH, was measured by asking: ‘Today, I was distressed by the voices’ (7-point Likert scale from 1 = ‘not at all’ to 7 = ‘very’). The outcome variable for hypothesis 2b, subjectively experienced negative impact of AVH on social functioning, was measured by asking: ‘Today, the voices hindered my functioning’ (7-point Likert scale). The outcomes for the secondary hypotheses were also measured by daily monitoring, see Table 1 Appendix B for details.

#### 2.6.3 Validated interview and questionnaires

Extra confirmation of the ESM variables used for the primary and secondary outcomes was done by comparison to validated interview and questionnaires. See Appendix B for an overview of the questionnaires and the specific items used.

### 2.7 Statistical analyses

Aiming for a small to medium effect size (f = 0.30), a power of 0.80, an α level of 0.05, and a drop-out rate of 20 %, in a repeated measures design a total of 50 participants per condition was needed. SPSS version 27 (IBM Corp, 2020) was used to compare group characteristics at baseline, using χ^2^ tests or ANOVA's. If a characteristic proved to be significantly different between the groups, this variable was included in all analyses.

Because of multiple testing, chances of type I errors were increased. Our power calculation was based on the detection of an effect on our primary outcomes and in addition many other exploratory secondary outcomes were examined. VanderWeele & Marthur (VanderWeele and Mathur, 2019) argued the Bonferroni correction is, in this context, a too severe penalty and not in proportion with the accompanying increase of type 2 error. Therefore, we applied the Benjamini-Hochberg false discovery rate procedure to our primary outcomes, by using the ‘p-adjust BH’ command in R (R Core Team, 2020).

We planned to perform multilevel regression analyses on the ESM and DM data. However, the data did not comply with all assumptions of multilevel analysis. Therefore, more conservative testing by performing linear regression analysis in SPSS Version 27 (IBM Corp, 2020) was chosen. For every individual, a mean was calculated for every variable per measurement moment (baseline, post-intervention, and follow-up). Linear regression analyses were performed on the difference scores of the variables between post-intervention and baseline as the dependent variable (i.e. we subtracted the baseline score from the post-intervention score) and condition as the independent variable; if the effect on condition proved to be significant, we investigated whether this was maintained by performing linear regression analyses on the difference scores of follow-up and baseline. We applied an intention-to-treat approach.

Data of the interview and questionnaires were analyzed using linear regression analysis in SPSS (IBM Corp, 2020).

## 3 Results

**Fig. 2:**
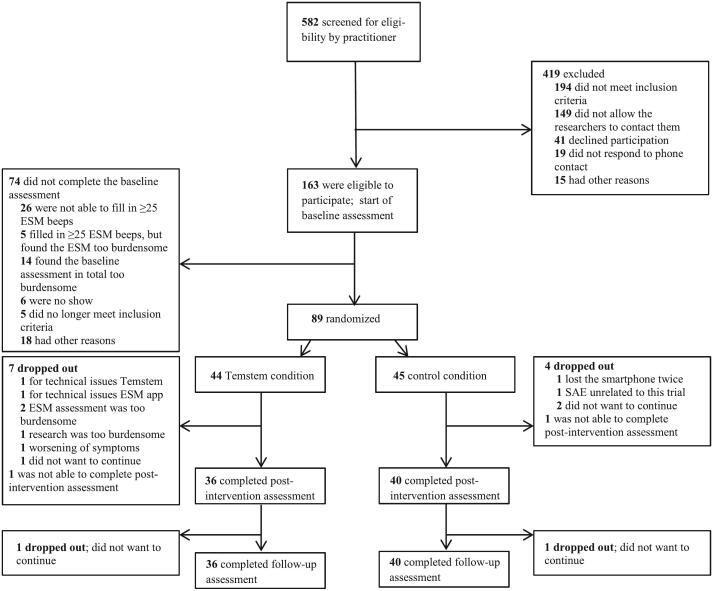
Participant flowchart.

Participants were recruited from March 2016 through April 2018. 44 Participants were randomized to the Temstem condition and 45 to the control condition (see Fig. 2). The groups did not significantly differ in demographical characteristics. There were no variables added as covariates in the analyses (see Table 1).

There were eight study dropouts in the Temstem condition and five in the control condition (see Fig. 2). In each condition one participant was not able to complete post-intervention assessment. Unfortunately, due to technical errors, the actual use of Temstem was only recorded for 24 out of 44 participants in the Temstem condition. Of these 24 participants, four participants did not fulfill the ‘Temstem user’ criteria (i.e. at least reaching level 2 of one of the games or having used Temstem for 15 times) and were intervention dropouts.

For changes in medication regiment per condition, see Appendix C.

### 3.1 Results

#### 3.1.1 Primary outcomes

**Fig. 3:**
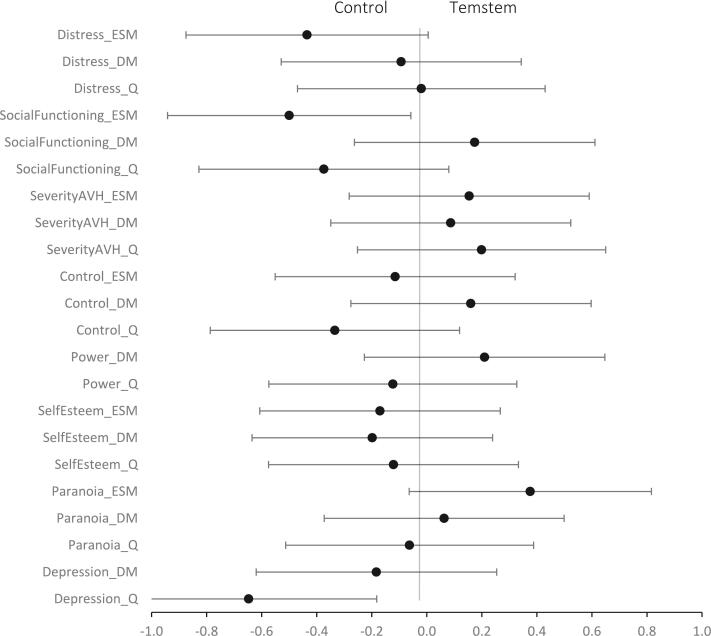
Forestplot of Cohen's d per outcome at post-intervention. *Note: Effect sizes are based on the weighted means. ESM = Exprience Sampling Method; DM = Daily Monitoring; Q = Questionnaire.

At post-intervention, there was no significant difference between the groups on momentary distress, *F*(2,78) = 3.835, p^adj^ = 0.108, subjective experienced voice hearing distress, *F*(1,79) = 0.173, p^adj^ = 0.678, social functioning, *F*(1,79) = 4.838, p^adj^ = 0.108, and subjectively experienced impediment of functioning by the voices, *F*(1,79) = 0.642, p^adj^ = 0.566 (see Table 2 and Fig. 3).

**Table 2:** Weighted means and standard deviations for primary and secondary outcome measures in the intervention and control groups.

Outcome	Measurement	Temstem group			Control group		
		Baseline	Post-treatment	Follow-up	Baseline	Post-treatment	Follow-up
Primary outcomes							
Momentary distress	ESM	3.08 (1.31)	3.12 (1.37)	3.16 (1.45)	2.92 (1.14)	2.74 (1.15)	2.68 (1.15)
Subjective experienced AVH distress	DM	4.13 (1.79)	3.83 (1.95)	3.39 (1.70)	3.49 (1.61)	3.18 (1.82)	3.29 (2.01)
Social functioning	ESM	0.62 (0.20)	0.56 (0.26)	0.59 (0.28)	0.52 (0.23)	0.52 (0.25)	0.47 (0.25)
Subjective experienced impediment of functioning by AVH	DM	4.20 (1.77)	3.69 (1.94)	3.34 (1.73)	3.63 (1.42)	3.40 (1.77)	3.23 (1.92)

Secondary outcomes							
Severity of AVH	ESM	4.95 (1.96)	4.51 (2.25)	4.22 (2.10)	4.29 (1.75)	4.01 (2.01)	4.16 (2.11)
Severity of AVH	DM	5.22 (1.55)	4.62 (1.88)	4.19 (1.82)	4.92 (1.42)	4.47 (1.68)	4.33 (1.85)
Momentary control	ESM	4.68 (1.14)	4.46 (1.48)	4.61 (1.56)	4.23 (1.17)	4.40 (1.38)	4.56 (1.51)
Control over AVH	DM	3.74 (1.48)	3.97 (1.64)	4.22 (1.45)	3.79 (1.50)	3.77 (1.84)	3.42 (2.06)
Power in relation to AVH	DM	3.36 (1.55)	3.85 (1.64)	3.95 (1.53)	3.75 (1.58)	3.85 (1.87)	3.57 (2.01)
Momentary self-satisfaction	ESM	4.11 (1.68)	3.94 (1.62)	3.86 (1.73)	4.05 (1.62)	4.16 (1.78)	4.41 (1.77)
Self-esteem	DM	3.77 (1.49)	3.79 (1.66)	3.93 (1.67)	3.77 (1.54)	4.03 (1.85)	4.13 (1.81)
Momentary paranoia	ESM	2.75 (1.68)	2.60 (1.68)	2.76 (1.85)	2.85 (1.58)	2.97 (1.80)	2.84 (1.71)
Paranoid ideation	DM	2.65 (1.64)	2.60 (1.74)	2.92 (1.78)	3.02 (1.80)	3.12 (1.92)	3.23 (1.82)
Depression	DM	3.66 (1.62)	3.59 (1.75)	3.37 (1.65)	3.37 (1.46)	3.19 (1.75)	2.90 (1.77)

Validation of outcomes							
AVH distress	AHRS	2.80 (0.88)	2.39 (1.18)	2.30 (1.08)	2.71 (0.79)	2.30 (0.94)	2.28 (0.99)
Social functioning	SDS	18.27 (8.01)	16.64 (7.71)	15.94 (7.29)	18.91 (5.34)	14.55 (7.12)	14.93 (7.37)
Severity of AVH	AHRS	3.22 (0.76)	2.70 (1.22)	2.58 (1.17)	3.07 (0.62)	2.80 (0.95)	2.93 (0.94)
Control over AVH	AHRS	3.36 (0.97)	3.31 (1.17)	3.43 (0.96)	3.67 (0.64)	3.18 (1.26)	3.32 (1.41)
Power in relation to AVH	BAVQ-R	17.86 (4.33)	16.64 (5.06)	16.28 (5.11)	17.33 (4.33)	15.95 (3.97)	16.03 (3.54)
Self-esteem	SERS-SF	7.16 (24.90)	5.11 (24.40)	3.69 (22.97)	0.09 (25.51)	3.68 (25.95)	2.81 (26.10)
Paranoid ideation	GPTS	76.04 (32.52)	74.28 (30.07)	72.28 (32.49)	77.82 (28.49)	75.45 (29.97)	73.82 (32.53)
Depression	BDI-II	25.50 (15.51)	27.51 (14.92)	24.72 (15.92)	21.33 (11.87)	19.88 (11.93)	20.92 (14.06)

Note: ESM = Experience Sampling Method; DM = daily monitoring; AHRS = Auditory Verbal Hallucination Scale; SDS = Sheehan Disability Scale; BAVQ-R: Beliefs About Voices Questionnaire – Revised; SERS-SF: Self-Esteem Rating Scale – Short Form; GPTS: Green et al. Paranoid Thoughts Scale; BDI-II: Beck Depression Inventory – Second Edition.

#### 3.1.2 Secondary outcomes

In contrast to our hypothesis, momentary control, as measured by ESM, was temporarily significantly decreased in the Temstem condition compared to the control condition at post-intervention *F*(2,78) = 4.535, *p* = .036, although this was not maintained at follow-up, *F*(2,69) = 1.549, *p* = .217. Control over voices as measured by daily monitoring, did not differ between groups at post-intervention, *F*(1,79) = 0.505, *p* = .479. Furthermore, none of the other outcomes differed significantly between the conditions (see Table 2 Appendix B and Fig. 3).

#### 3.1.3 Validation of primary and secondary outcomes

The non-significant findings were validated by the interview and questionnaires, except for depression; opposed to our hypothesis, depression symptoms as measured by a questionnaire were temporarily significantly increased in the Temstem condition in comparison to the control group (F(1.73) = 7.803, *p* = .007), but this effect was not maintained at follow-up (F(1.72) = 0.136, *p* = .714). Control over voices as measured by an interview did not differ between the groups at post-intervention, *F*(1,74) = 2.078, *p* = .154 (see Table 2 Appendix B and Fig. 3).

### 3.2 Technical issues

During the trial, technical issues of both Temstem and the ESM app were common and affected the study. At least part of these participants reported that these issues were burdensome, although we did not record how this affected their mood and motivation. See Appendix D for the reported issues.

## 4 Discussion

With the Temstem Randomized Controlled Trial (RCT), we investigated whether the stand-alone use of the Temstem app plus AVH monitoring reduced voice hearing distress and improved social functioning as compared to AVH monitoring only in a clinical voice hearing sample. Reported effects of Temstem on severity of voices, control over and power in relation to voices, self-esteem, depression, and paranoid ideation, were compared by a validated interview and questionnaires. We did not find any significant differences between the two conditions on primary outcomes. The secondary outcomes were also largely non-significant, except for momentary control measured by ESM; momentary control was temporarily lowered in the Temstem condition compared to the control condition at post-intervention, although this effect was not maintained at follow-up. In addition, the non-significant effects were validated by an interview and questionnaires; according to a questionnaire, only depression was temporarily significantly increased in the Temstem condition compared to the control condition, but this effect was not maintained at follow-up. We conclude that, compared to monitoring, there is no evidence that stand-alone use of the Temstem app is effective in reducing voice hearing distress and in improving social functioning in individuals with distressing and frequent voices.

The findings are not in line with our expectations. Our naturalistic study, that involved data of 1.048 Temstem users, showed that voice hearing distress was significantly decreased after a Silencing session, and emotionality and vividness of voice memories were significantly lowered after a Challenging session (Jongeneel et al., 2022). Obviously, the lack of a control condition in that study limits the interpretability of the results. However, playing a language game while hearing voices, on which Silencing is based on, was previously found to be effective in suppressing voices (Bick and Kinsbourne, 1987; Erickson and Gustafson, 1968; Green and Kinsbourne, 1989). In addition, dual tasking, which Challenging is based on, was found to be effective in reducing voice memory emotionality, vividness, and credibility (Jongeneel et al., 2020; Matthijssen et al., 2019).

A potential explanation for non-effectiveness of Temstem in this trial, can be found in the data of a short reflective questionnaire. Participants stated that they used Temstem mostly at home (57 %) and when being alone (58 %), while an important aim of Temstem is to cope with voices at moments that individuals are outside and/or with others. We believe that using Temstem at such moments might help to experience less voice hearing distress in social situations, therefore improving social functioning on the long term. By using Temstem mostly alone at home, social functioning as we measured could not be directly improved.

In addition, the registration data of the app (whereby only the data of 24 out of 44 participants was registered due to technical difficulties) showed that Challenging was less used than Silencing (about 1:2). This is in line with the outcomes of our naturalistic study, in which we observed that Challenging was used in 24 % of the sessions (Jongeneel et al., 2022). A potential explanation for less engagement with Challenging might be that this function is more difficult to use due to the dual tasking mechanism (i.e. thinking about a negative voice hearing statement while playing a language game). Also, Silencing is used when the voices are present. Challenging can best be used when the voices are absent or in the background. Without the trigger of hearing voices in time, it may be more difficult for the users to remind themselves to use the app for this purpose.

Offering prompts might trigger people to engage with Challenging. Actissist, an unguided app for persons with psychosis, was tested in a small RCT (Bucci et al., 2018). Actissist significantly improved negative symptoms, general and total psychosis symptoms and mild to moderate depression symptoms, although these effects were lost at 22-week follow-up. An important difference between Actissist and Temstem is that Actissist users received a prompt multiple times a day to remind them using the app. A secondary analysis of the Actissist pilot trial data showed that engagements with the app were in 87 % of cases app-initiated rather than self-initiated (Eisner et al., 2023). Temstem was also designed to prompt the participant once a day, but due to technical error this function did not work. In their systematic review, Free et al. (Free et al., 2013) concluded that health apps whereby the participants were supported and reminded of using the app by receiving text messages, were more effective than interventions without these messages. It might be essential to fix this function in order to improve potential effectiveness, which could be further investigated in a fractorial/factorial design (Collins et al., 2005).

Temstem was offered in an unguided manner in the RCT. Unguided apps have several advantages; for example, unguided apps can be widely deployed and small effects may lead to important health gains; and they are repeatedly available, while guided apps are dependent on therapist time. Nonetheless, guidance to some degree might be beneficial for users. SAVVy (Smartphone-Assisted coping focused interVention for Voices; (Bell et al., 2018; Bell et al., 2020) is a blended intervention for voice hearing individuals, based on Ecological Momentary Assessment and Intervention (EMA/I). Participants received an app and four face-to-face sessions with a therapist. Although SAVVy did not have a significant effect on overall voice severity in a pilot trial, it significantly increased awareness of voice patterns and coping with voices (Bell et al., 2020). Also, a pilot study has been performed wherein persons with voices used a self-help workbook and received additional guidance of a therapist with a maximum of 8 sessions (the Guided self-help cognitive behavioral intervention for VoicEs, GiVE; (Hazell et al., 2016)). Results showed that 88 % of the participants engaged with at least 6 out of 8 sessions and compared to treatment-as-usual and supportive counselling, negative voice impact and distress were decreased in the GiVE arm (Hayward et al., 2021). In addition, meta-analyses showed that in clinical patients with a variety of diagnoses, guided Internet-based mental health interventions were significantly more effective than unguided interventions (Baumeister et al., 2014; Karyotaki et al., 2021). It might be that using Temstem under guidance of a therapist (to some degree) results in an effective tool in patients with distressing and frequent voices.

It is unclear how to interpret the (temporarily) negative findings of decreased momentary control, measured by ESM, at post-treatment and increased depression, measured by a validated questionnaire, at post-treatment in the Temstem condition compared to the control condition. There are multiple potential explanations, i.e. disappointment or frustration that people may have experienced when they discovered Temstem did not help or function as expected; chance finding because of multiple testing; measurement characteristics; or differences between the diagnoses in the two study arms. We will look at this in more detail later.

### 4.1 Strengths and limitations

This study had several strengths. First, we were able to include 89 voice hearing individuals with severe mental health difficulties, despite the heavy task load and the severity of the participants' voices. Second, the attrition rate, which indicates how many individuals were unable to complete the trial assessments, was 14.6 % for both post-intervention and follow-up. This was lower than Linardon & Fuller-Tyszkiewicz (Linardon and Fuller-Tyszkiewicz, 2020) in their systematic and meta-analytic review about attrition rates in smartphone delivered interventions reported; they showed average rates of 24.1 % at post-intervention and 35.5 % at follow-up.

There are multiple limitations of this study that are important to report. Momentary distress and momentary control were probable too generic measures, assessing general daily life distress and control. Since the study sample consisted of individuals with problems in multiple areas of life, these outcomes were not sufficient to measure changes in distress or control specifically related to voice hearing. Also, social functioning was calculated by common used ESM items (i.e. activity and social company). However, there is no validated operationalization for measuring social functioning based on these items. Since the study sample was, on average, a fairly passive sample, with individuals who were mainly alone and/or at home, we choose to categorize ‘Relaxing-passive’ and ‘Eating and drinking’ as passive activities. Because of this, we have potentially miscategorized a few events and the reported data may be an underestimate of actual social functioning. Furthermore, we used combinations of AHRS items that do not reflect the factor structure for this measure (Woodward et al., 2014), since we were mainly interested in whether the data of the validated questionnaires were consistent with the ESM and/or daily monitoring data. This complicates the interpretation of findings and comparison with other studies.

Because of violation of several assumptions of multilevel regression analysis, we performed a more conservative testing. This linear regression analysis is not in accordance with our planned multilevel regression analysis as described in our protocol paper (Bucci et al., 2015). Our power analysis was based on multiple testing; our decision might have resulted in an underpowered study, making it less probable to assess small differences between the two studied arms.

The trial design was very intensive. For example, according to Delespaul (Delespaul, 1995), an ESM protocol should not last longer than two minutes per questionnaire. In our study it took participants on average 3 min to complete each questionnaire. The high ESM burden led to many individuals finding participation too burdensome, causing them not to be able or willing to participate in the RCT (42 % of eligible participants) or to drop-out during the trial (at least 23 % of all drop-outs).

The apps had technical failures. This increased the burden of participating in this trial further and in specific in the Temstem condition. Although participants reported increased stress levels by these issues, we did not record how this exactly influenced results.

### 4.2 Clinical and research implications and considerations

After the small pilot study (unpublished), we moved to an RCT whereby persons with lived experience were no longer involved. These choices were pragmatic, partially based on the minimal funding the project received. Receiving funding specifically for app development and maintenance is difficult in the Netherlands. Organizations that fund scientific research of eHealth applications, often exclude funds for app development. More thorough usability testing and involvement of voice hearing individuals in the development of the RCT, would have potentially resulted into higher app engagement; an improved, bug free version of Temstem; other, improved operationalizations of outcomes such as distress and social functioning; and a lower study burden for participants (e.g. fewer ESM items). In future research, we aim to re-involve persons with lived experience in the processes of updating or redesigning Temstem, in the development of a guided protocol, and in the development of a mixed method study whereby quantitative and qualitative measures will be combined to study the optimal degree of guidance by a therapist, and the usability and effects of guided Temstem.

There are a few specific suggestions we believe are of importance to mention. First, recruiting participants for participation in our RCT was difficult and progressed slowly, partly because of the high study burden. It is recommended to first pilot test the study protocol to test whether the protocol is feasible. Also, it might be beneficial to consider other study designs that require less participants, such as a multiple baseline design. Second, an app is never ‘finished’. When the app is ‘live’, we recommend to monitor it continuously for bugs and technical issues by a technical support system. Third, we suggest researchers who develop an app to add prompts that remind users to use the app and to design it in a blended fashion. See also Weisel et al. (Weisel et al., 2019) and Alqahtani & Orji (Alqahtani and Orji, 2020). Fourth, we argue it is very valuable to store and analyze the user data gathered by the app. This can provide insight in the actual use. Fifth, the research protocol should be adjusted to the cognitive and motivational limitations of the population. For example, ESM questionnaires should not exceed the two-minute timeframe.

### 4.3 Conclusions

In a sample of daily voice hearing individuals with severe mental illness, stand-alone use of the Temstem app was not found to be effective in reducing voice hearing distress and improving social functioning, as compared to symptom monitoring. In order to potentially improve effectiveness of an mHealth tool in a population of people with frequent and distressing voices, we recommend to involve persons with lived experience in all stages of development, usability testing, and effectiveness research; to thoroughly test the (technological) usability before the start of an RCT; to test to what degree guidance of a therapist is needed to optimize effectiveness (for example: 1 short meeting to explain the app, to 8 sessions of 60 min of face to face therapy); and to remind the user to actually use the tool by providing prompts.

## Financial support

The Temstem trial was funded by a grant awarded to DvdB and MvdG by the ‘Innovatie Platform Parnassia’ (Monsterseweg 93, 2553 RJ Den Haag), and this project received financial support of the Amsterdam UMC, department of Clinical Psychology.

## Declaration of competing interest

None.

## Appendix A Supplementary data

Supplementary material Image 1
