# Supplementary material for: Effects on voice hearing distress and social functioning of unguided application of a smartphone app — A randomized controlled trial
Source: Internet Interv. 2024 Jan 26;35:100717. doi: 10.1016/j.invent.2024.100717 (PMC10847757; doi:10.1016/j.invent.2024.100717)
Supplement: Supplementary file 1 — Supplementary material [file mmc1.docx]

**Appendix**

1. **Co-creation process**

In 2011 a small innovation project called Recovery from Psychosis by Design was conducted by Parnassia Group and the Technical University Delft. Voice hearing individuals receiving mental health care, designers, clinicians, and scientists, conversed about the needs, wishes, values, and interests of the voice hearers into supporting their mental health. Hereafter, literature research was performed, and several small iterative tests were explored with mock-ups. During this process, voice hearing individuals were actively involved. The concept of developing an app emerged gradually. All results of the discussions, tests, and literature research led to the design of Temstem, which included language games and science-based working mechanisms. By continuously involving voice hearing individuals with need for care (or ‘users’), Temstem was built and tested by all stakeholders in an iterative process. This resulted in the first Temstem app version. This version was tested by 8 persons with voices on usability and feasibility. The results of this pilot research were positive (unpublished). Hereafter, we performed a Randomized Controlled Trial (RCT). In the development and execution of this RCT, persons with lived experience were no longer involved. Since then, however, we have adapted this policy in our research group, and all our projects now involve lived experience experts in all project phases.

1. **Secondary outcomes**

*Secondary hypotheses:*

Hypothesis 3: The severity of AVH will decrease after using Temstem for 5 weeks, as measured by ESM/daily monitoring.

Hypothesis 4: Temstem will enhance control over AVH, as measured by ESM/daily monitoring.

Hypothesis 5: Temstem will enhance power in relation to AVH, as measured by daily monitoring.

Hypothesis 6: Temstem will improve self-esteem, depression and/or paranoid ideation as measured by ESM/ daily monitoring.

Hypothesis 7: The outcomes of hypotheses 1–6 will be confirmed by validated interviews and/or questionnaires.

*Validated interview and questionnaires*

For AVH distress, severity of AVHs, and control over AVHs the Auditory Hallucinations Rating Scale (AHRS; Haddock, McCarron, Tarrier, & Faragher, 1999) was used; for social functioning the Sheehan Disability Scale (SDS; Sheehan & Sheehan, 2008) was used; to measure power of AVHs the Beliefs About Voices Questionnaire-Revised (BAVQ-R; Chadwick, Lees, & Birchwood, 2000) was included; self-esteem was measured by the Self-Esteem Rating Scale-Short Form (SERS-SF; Lecomte, Corbière, & Laisné, 2006); the Green et al. Paranoid Thoughts Scale (GPTS; Green et al., 2008) was used to measure paranoid ideation; and the Beck Depression Inventory, Second Edition (BDI-II; Beck, Steer, Ball, & Ranieri, 1996) was included to measure depression. See Table 1 in Appendix A for the specific items. All questionnaires were conducted at baseline, post-intervention, and follow-up. For information about the psychometric properties, see our protocol paper (Jongeneel et al., 2018).

| **Hypothesis**  *Table 1. Operationalisation of secondary outcomes* | **Outcome** | **Measurement instrument** | **Item** | **Measuring range** |
| --- | --- | --- | --- | --- |
| **Secondary** |  |  |  |  |
| 3 | Severity of AVH | ESM | I’m hearing voices | 1 (not at all) – 7 (very) |
| 3 | Severity of AVH | DM | Today, I heard voices/The voices were annoying^a^ | 1 (not at all) – 7 (very) |
| 4 | Momentary control | ESM | I’m in control | 1 (not at all) – 7 (very) |
| 4 | Control over AVH | DM | Today, I had control over the voices | 1 (not at all) – 7 (very) |
| 5 | Power in relation to AVH | DM | Today, I was more powerful than the voices | 1 (not at all) – 7 (very) |
| 6 | Momentary self-satisfaction | ESM | I am satisfied with myself as a person | 1 (not at all) – 7 (very) |
| 6 | Self-esteem | DM | Today, I had a positive sense of self-worth | 1 (not at all) – 7 (very) |
| 6 | Momentary paranoia | ESM | I’m suspicious | 1 (not at all) – 7 (very) |
| 6 | Paranoid ideation | DM | Today, I was suspicious | 1 (not at all) – 7 (very) |
| 6 | Depression | DM | Today, I had little interest or pleasure in activities/I’ve felt low, depressive, or hopeless^a^ | 1 (not at all) – 7 (very) |
| **Validation** |  |  |  |  |
| 7 | AVH distress | AHRS | Item 9;  Intensity of suffering by AVHs | 0 (not at all) – 4 (very) |
| 7 | Social functioning | SDS | Total score of 3 items: work, social life, home life | 0 (not at all) – 30 (extremely) |
| 7 | Severity of AVH | AHRS | Item 1, 6 and 7;  Frequency of AVHs/ Negative content/ Severity of negative content of AVHs^a^ | 0 (not at all) – 4 (very) |
| 7 | Control over AVH | AHRS | Item 11;  Control over AVHs | 0 (always) – 4 (never) |
| 7 | Power in relation to AVH | BAVQ-R | Subscale omnipotence/power, 6 items | 6 (voice has no power) – 24 (voice has all power) |
| 7 | Self-esteem | SERS-SF | Totalscore positive self-esteem (10 items) – negative self-esteem (10 items) | -70 (negative self-esteem) – 70 (positive self-esteem) |
| 7 | Paranoid ideation | GPTS | Totalscore of 32 items | 32 (not at all) – 160 (very) |
| 7 | Depression | BDI-II | Totalscore of 21 items | 0 (not at all) – 63 (very) |

Note: ^a^ the mean of these items was calculated. ESM = Experience Sampling Method; DM = daily monitoring; AHRS = Auditory Verbal Hallucination Scale; SDS = Sheehan Disability Scale; BAVQ-R: Beliefs About Voices Questionnaire – Revised; SERS-SF: Self-Esteem Rating Scale – Short Form; GPTS: Green et al. Paranoid Thoughts Scale; BDI-II: Beck Depression Inventory – Second Edition.

*Table 2. Results of secondary outcomes*

| **Outcome** | **Post-intervention** | |  | **Follow-up** |  | |  | |
| --- | --- | --- | --- | --- | --- | --- | --- | --- |
|  | df | F | p | df | F | p | |  |
| **Experience Sampling Method** | |  |  |  |  |  | |  |
| Severity of AVH | 1,79 | 0.478 | 0.492 | 1,70 | 2.656 | 0.108 | |  |
| Momentary control | 2,78 | 4.111 | 0.046 | 2,69 | 1.235 | 0.270 | |  |
| Momentary self-satisfaction | 1,79 | 0.570 | 0.452 | 1,70 | 0.024 | 0.877 | |  |
| Momentary paranoia | 1,79 | 2.949 | 0.090 | 1,70 | 5.472 | 0.022 | |  |
| **Daily monitoring** |  |  |  |  |  |  | |  |
| Severity of AVH | 1,79 | 0.164 | 0.686 | 1,65 | 1.969 | 0.165 | |  |
| Control over AVH | 1,79 | 0.505 | 0.479 | 1,65 | 6.032 | 0.017 | |  |
| Power in relation to AVH | 1,79 | 0.886 | 0.350 | 1,65 | 2.858 | 0.096 | |  |
| Self-esteem | 1,79 | 0.748 | 0.390 | 1,65 | 0.189 | 0.665 | |  |
| Paranoid ideation | 1,79 | 0.077 | 0.782 | 1,65 | 0.021 | 0.885 | |  |
| Depression | 1,79 | 0.645 | 0.424 | 1,65 | 2.279 | 0.136 | |  |
| **Questionnaires** |  |  |  |  |  |  | |  |
| Distress | 1, 74 | 0.155 | 0.695 | 1, 75 | 1.754 | 0.189 | |  |
| Social functioning | 1, 74 | 2.629 | 0.109 | 1, 75 | 0.269 | 0.605 | |  |
| Severity of AVH | 1, 74 | 0.791 | 0.377 | 1, 75 | 5.592 | 0.021 | |  |
| Control over AVH | 1, 74 | 2.078 | 0.154 | 1, 75 | 2.451 | 0.122 | |  |
| Power in relation to AVH | 1, 74 | 0.294 | 0.589 | 1, 74 | 0.298 | 0.587 | |  |
| Self-esteem | 1, 73 | 0.271 | 0.604 | 1, 72 | 1.063 | 0.306 | |  |
| Paranoid ideation | 1, 74 | 0.098 | 0.755 | 1, 72 | 1.360 | 0.247 | |  |
| Depression | 1, 73 | 7.803 | 0.007 | 1, 72 | 0.136 | 0.714 | |  |

1. **Medication changes**

In the Temstem condition, 3 changes in medication during the trial were reported: one AP dose increased, there was one change of AP medication brand, and one person started with AP medication during the study. There were no changes in AD medication reported. In the control group, there were 10 changes in in antipsychotic (AP) and/or antidepressant (AD) medication reported: one dose of AP medication was lowered, one stopped with AP medication, four persons changed their AP medication brand, one increased AP and started with AD medication, and three persons stopped with AD medication.

**D. Technical issues**

D1. Technical issues with the Temstem app

Seven out of 33 participants (21.2%) in the Temstem condition that filled in the Temstem usability questionnaire at post-intervention reported technical problems (see Table 4). Since the usability questionnaire concerned self-report, we assume the reported issues are an underestimation of the actual number of issues. Besides the reported issues, the Reflection function, whereby participants should have received a prompt reminding them to fill in a very short questionnaire about their voices, did not work. This issue could not be solved. One person dropped out explicitly because of technical issues with Temstem.

*Table 3. Issues of the Temstem app*

| **Issue** | **N(%)^a^** |
| --- | --- |
| Lingo Tapper crashed one or more times  Lingo Tapper disappeared several times while playing  Word Link did not work correctly  Temstem sometimes did not work correctly  Once receiving an error while opening the app  **Total** | 2 (6.1)  1 (3.0)  2 (6.1)  1 (3.0)  1 (3.0)  **7 (21.1)** |

^a^33 persons out of 44 filled in the Temstem questionnaire

D2. Technical issues with the ESM app

The ESM app initially worked on all our different research-smartphones. However, an increasing amount of technical issues (such as not receiving prompts) made us test various smartphones and buy multiple phones of one specific brand what initially resolved the issues. However, after an Android update, these phones automatically switched to a battery saving mode, causing the ESM app not to send prompts anymore. After finding a workaround, most technical difficulties were overcome, however this workaround was no assurance the app would work smoothly. Several participants still reported minor to major difficulties. After each ESM period, we asked participants whether they encountered any difficulties or issues with this app; respectively 17, 21, and 12 participants reported (a) (severe) technical issue(s) with the ESM app at baseline, post-intervention, and follow-up. One person dropped out of the study due to technical problems with the ESM app. For the specific reported issues, see Table 5.

*Table 4. Issues of the ESM app*

| **Issue** | **Baseline** | **Post-intervention** | **Follow-up** |
| --- | --- | --- | --- |
|  | N (%) | N (%) | N (%) |
| No or less prompts  Crashing of the app  Receiving a prompt, but no questionnaire appears  Other  **Total** | 5 (5.6)  3 (3.4)  7 (7.9)  2 (2.2)  **17 (19.1)** | 5 (6.6)  1 (1.3)  8 (10.5)  7 (9.2)  **21 (27.6)** | 1 (1.3)  3 (3.9)  4 (5.2)  4 (5.2)  **12 (15.8)** |
